# In Vitro Interaction of AB-FUBINACA with Human Cytochrome P450, UDP-Glucuronosyltransferase Enzymes and Drug Transporters

**DOI:** 10.3390/molecules25194589

**Published:** 2020-10-08

**Authors:** Sunjoo Kim, Dong Kyun Kim, Yongho Shin, Ji-Hyeon Jeon, Im-Sook Song, Hye Suk Lee

**Affiliations:** 1 Drug Metabolism and Bioanalysis Laboratory, College of Pharmacy, The Catholic University of Korea, Bucheon 14662, Korea; sjkim712@catholic.ac.kr (S.K.); kdk3124@catholic.ac.kr (D.K.K.); driger6103@catholic.ac.kr (Y.S.); 2 College of Pharmacy and Research Institute of Pharmaceutical Sciences, Kyungpook National University, Daegu 41566, Korea; kei7016@naver.com

**Keywords:** AB-FUBINACA, drug–drug interaction, cytochrome P450, uridine 5′-diphospho-glucuronosyltransferases, drug transporters

## Abstract

AB-FUBINACA, a synthetic indazole carboxamide cannabinoid, has been used worldwide as a new psychoactive substance. Because drug abusers take various drugs concomitantly, it is necessary to explore potential AB-FUBINACA-induced drug–drug interactions caused by modulation of drug-metabolizing enzymes and transporters. In this study, the inhibitory effects of AB-FUBINACA on eight major human cytochrome P450s (CYPs) and six uridine 5′-diphospho-glucuronosyltransferases (UGTs) of human liver microsomes, and on eight clinically important transport activities including organic cation transporters (OCT)1 and OCT2, organic anion transporters (OAT)1 and OAT3, organic anion transporting polypeptide transporters (OATP)1B1 and OATP1B3, P-glycoprotein, and breast cancer resistance protein (BCRP) in transporter-overexpressing cells were investigated. AB-FUBINACA inhibited CYP2B6-mediated bupropion hydroxylation via mixed inhibition with *K_i_* value of 15.0 µM and competitively inhibited CYP2C8-catalyzed amodiaquine *N*-de-ethylation, CYP2C9-catalyzed diclofenac 4′-hydroxylation, CYP2C19-catalyzed [*S*]-mephenytoin 4′-hydroxylation, and CYP2D6-catalyzed bufuralol 1′-hydroxylation with *K_i_* values of 19.9, 13.1, 6.3, and 20.8 µM, respectively. AB-FUBINACA inhibited OCT2-mediated MPP^+^ uptake via mixed inhibition (*K_i_*, 54.2 µM) and competitively inhibited OATP1B1-mediated estrone-3-sulfate uptake (*K_i_*, 94.4 µM). However, AB-FUBINACA did not significantly inhibit CYP1A2, CYP2A6, CYP3A4, UGT1A1, UGT1A3, UGT1A4, UGT1A6, or UGT2B7 enzyme activities at concentrations up to 100 µM. AB-FUBINACA did not significantly inhibit the transport activities of OCT1, OAT1/3, OATP1B3, P-glycoprotein, or BCRP at concentrations up to 250 μM. As the pharmacokinetics of AB-FUBINACA in humans and animals remain unknown, it is necessary to clinically evaluate potential in vivo pharmacokinetic drug–drug interactions induced by AB-FUBINACA-mediated inhibition of CYP2B6, CYP2C8, CYP2C9, CYP2C19, CYP2D6, OCT2, and OATP1B1 activities.

## 1. Introduction

Synthetic cannabinoids (SCs) are new psychoactive substances mimicking Δ9-tetrahydrocannabinol (THC), the active component of cannabis, and typically bind to the cannabinoid receptor type 1 (CB1) or type 2 (CB2) [1]. SC misuse has increased worldwide; 169 SCs are monitored by the European Monitoring Centre for Drugs and Drug Addiction (EMCDDA) via the EU Early Warning System established in December 2016.

**Figure 1:**
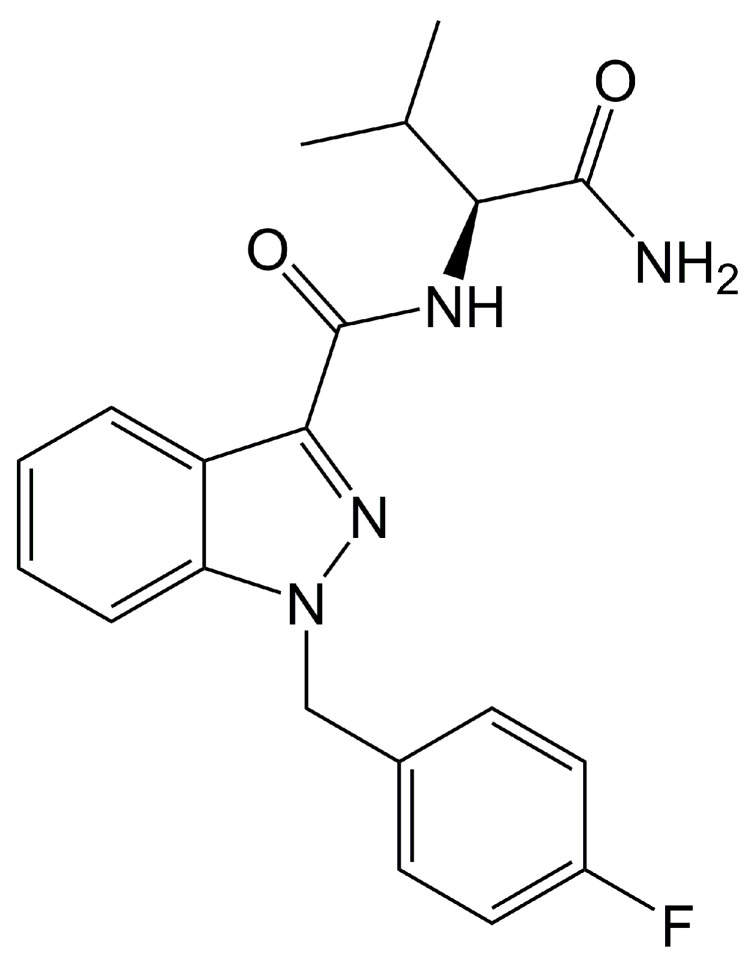
Chemical structure of AB-FUBINACA.

AB-FUBINACA (*N*-[(1*S*)-1-(aminocarbonyl)-2-methylpropyl]-1-[(4-fluorophenyl)methyl]-1H-indazole-3-carboxamide) is an SC (an indazole carboxamide) (Figure 1) exhibiting CB1 and CB2 agonist activities with EC_50_ values of 1.8 and 3.2 nM, respectively [2]. AB-FUBINACA was developed by Pfizer in 2009 as an analgesic drug candidate, but development was not pursued. The material was identified for the first time in Japanese herbal smoking blends in 2012 and has been included in Schedule I of the Controlled Substances Act by the US Drug Enforcement Administration since 2014 [3,4]. Use is controlled nationally in Germany, China, and Canada; and by the United Nations Office on Drugs and Crime.

AB-FUBINCA induced acute neurological disorders such as hyperreflexia, sensorimotor alterations, and spontaneous aggressiveness in mice when administered intraperitoneally at a single dose of 6 mg/kg [5]. In humans, the symptoms reported after intoxication of SCs are tachycardia, agitation, drowsiness, and vomiting etc. [4]. Repeated exposure of SCs leads to cause serious adverse reaction including coma and death. Among the cases of severe toxic effects and deaths associated with SCs reported from 2012 to 2015, the most prevalent cases were AB-CHIMICA and cases related to AB-FUBINACA, ADB-FUBINACA, AM2201, and JWH-018 were also reported [6,7]. In addition, metabolites of SCs such as JWH-018 and JWH-073 often retain higher affinity to CB1 or CB2 than THC [8]. Given the toxic events and increasing use of SCs and retained activity of SC metabolites, determination of SCs and their metabolites in serum, urine, hepatocytes, and liver microsomes has been developed [7]. AB-FUBINACA is extensively metabolized via hydrolysis of the amide group carboxylesterases 1 (CES1) and CES2, hydroxylation of the amino-oxobutane moiety and imidazole ring, defluorobenzylation and dehydrogenation by cytochrome P450s (CYPs) 2C19, 3A4, and 3A5, glucuronidation, and combinations thereof ([9,10,11,12], our unpublished data). The major metabolites of AB-FUBINACA identified after human liver microsomal incubation are different significantly from those in authentic human urine samples [9]. Hydrolysis of the amide group by carboxylesterases 1 (CES1) and CES2 and hydroxylation of the amino-oxobutane moiety are major metabolites found in urine and microsome samples [9,10] but hydroxylation of an imidazole ring and its glucuronide metabolite were mainly identified in intoxicated human urine samples [9].

Drug abusers frequently take several drugs concomitantly [13,14,15]; it is thus necessary to investigate the inhibitory effects of drugs on major drug-metabolizing enzymes such as cytochrome P450s (CYPs) and 5′-diphospho-glucuronosyltransferases (UGTs); and on clinically important drug transporters such as solute carrier transporters including the organic cation transporters (OCT)1, and OCT2, the organic anion transporters (OAT)1 and OAT3, the organic anion transporting polypeptides (OATP)1B1 and OATP1B3, and efflux transporters such as P-glycoprotein (P-gp) and breast cancer-resistance protein (BCRP) [16,17,18]. The inhibitory effects of phytocannabinoids including THC, cannabinol, cannabidiol [19,20,21,22,23,24,25], and synthetic cannabinoids (JWH-019, STS-135, UR-144, AM-2201, MAM-2201, EAM-2201, and APINACA) [4,26,27,28,29,30], on the major CYP and UGT enzymes or human liver microsomes, or recombinant CYP and UGT enzymes, have been reported [19,20,21,22,23,24,25,26,27,28,29,30].

Various drugs of abuse engage in transporter-mediated drug–drug interactions (DDIs); buprenorphine, norbuprenorphine, ibogaine, methadone, and THC-inhibited P-gp of HEK293-MDR1 cells; buprenorphine, norbuprenorphine, and ibogaine inhibited BCRP of HEK293-BCRP cells [31]; THC, cannabinol, cannabidiol, JWH-200, and WIN-55,212-2 inhibited BCRP ATPase activity [32,33]; and elaucine, JWH-200, mitragynine, and WIN-55,212-2 inhibited P-gp of Caco-2 cells [34]. The in vitro and in vivo inhibitory effects of AB-FUBINACA on major human drug-metabolizing enzymes, such as CYPs and UGTs, solute carrier transporters, and efflux transporters, remain unknown. We investigated the in vitro inhibitory effects of AB-FUBINACA on the activities of eight major CYPs and six UGTs of pooled human liver microsomes and on the transport activities of six solute carrier transporters and two efflux transporters of transporter-overexpressing cells, to predict potential AB-FUBINACA-induced DDIs.

## 2. Results

### 2.1. Inhibitory Effects of AB-FUBINACA on CYP and UGT Enzyme Activities of Human Liver Microsomes

**Figure 2:**
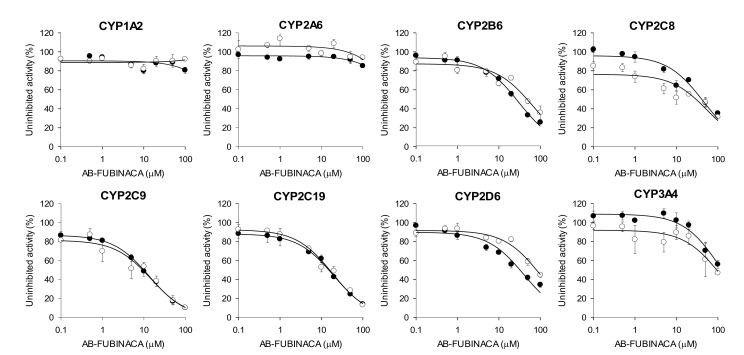
Inhibitory effects of AB-FUBINACA on CYP1A2-mediated phenacetin *O*-de-ethylation, CYP2A6-mediated coumarin 7-hydroxylation, CYP2B6-mediated bupropion hydroxylation, CYP2C8- mediated amodiaquine *N*-de-ethylation, CYP2C9-mediated diclofenac 4′-hydroxylation, CYP2C19-mediated [*S*]-mephenytoin 4′-hydroxylation, CYP2D6-mediated bufuralol 1′-hydroxylation, and CYP3A4-mediated midazolam 1′-hydroxylation by ultrapooled human liver microsomes with (◯) and without (⬤) 30-min preincubation with NADPH at 37 °C. The concentrations of cocktail CYP substrates were 50 μM phenacetin, 2.5 μM coumarin, 2.0 μM amodiaquine, 10 μM diclofenac, 100 μM [S]-mephenytoin, 5.0 μM bufuralol, 2.5 μM midazolam, and 50 μM bupropion. The data are means ± SDs (*n* = 3).

The reversible and time-dependent inhibitory effects (IC_50_ values) of AB-FUBINACA on eight major human CYP enzyme activities of ultrapooled human liver microsomes were evaluated using a cocktail of CYP substrates and liquid chromatography-tandem mass spectrometry (LC-MS/MS) (Figure 2 and Table 1). AB-FUBINACA inhibited CYP2B6-mediated bupropion hydroxylation, CYP2C8-mediated amodiaquine *N*-de-ethylation, CYP2C9-mediated diclofenac 4′-hydroxylation, CYP2C19-mediated [*S*]-mephenytoin 4′-hydroxylation, and CYP2D6-mediated bufuralol 1′-hydroxylation by ultrapooled human liver microsomes with IC_50_ values of 29.9, 44.3, 13.5, 19.7, and 40.4 μM, respectively (Figure 2 and Table 1). A 30-min preincubation of AB-FUBINACA with ultrapooled human liver microsomes and reduced β-nicotinamide adenine dinucleotide phosphate (NADPH) did not decrease the IC_50_ values of AB-FUBINACA for CYP2B6, CYP2C8, CYP2C9, CYP2C19, or CYP2D6 with a slight increase in CYP2B6, CYP2C8, and CYP2D6. It indicates that AB-FUBINACA inhibits the activities of CYP2B6, CYP2C8, CYP2C9, CYP2C19, or CYP2D6 in a concentration-dependent manner but it does not show time-dependent inhibition (Figure 2 and Table 1). AB-FUBINACA did not significantly inhibit CYP1A2-mediated phenacetin *O*-de-ethylation, CYP2A6-mediated coumarin 7-hydroxylation, or CYP3A4-mediated midazolam 1′-hydroxylation (IC_50_ >100 μM) by ultrapooled human liver microsomes with or without 30 min preincubation (Figure 2 and Table 1).

**Figure 3:**
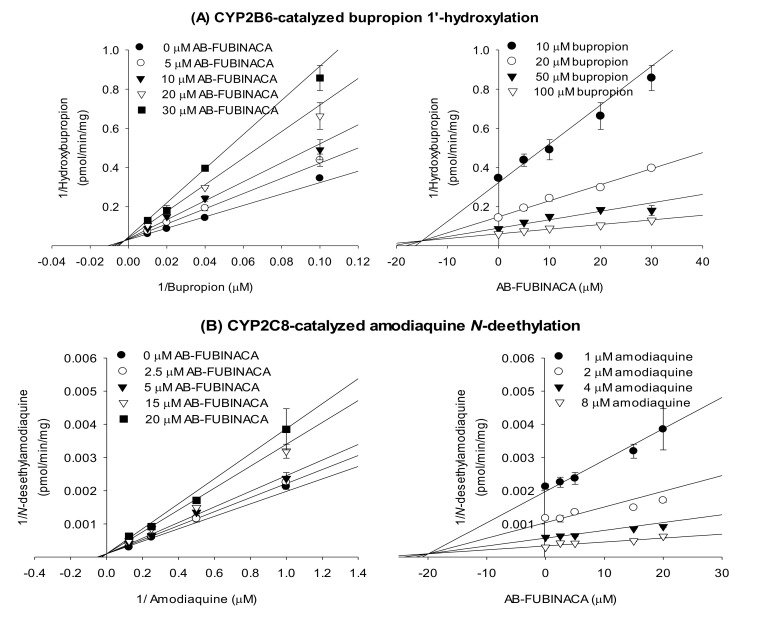

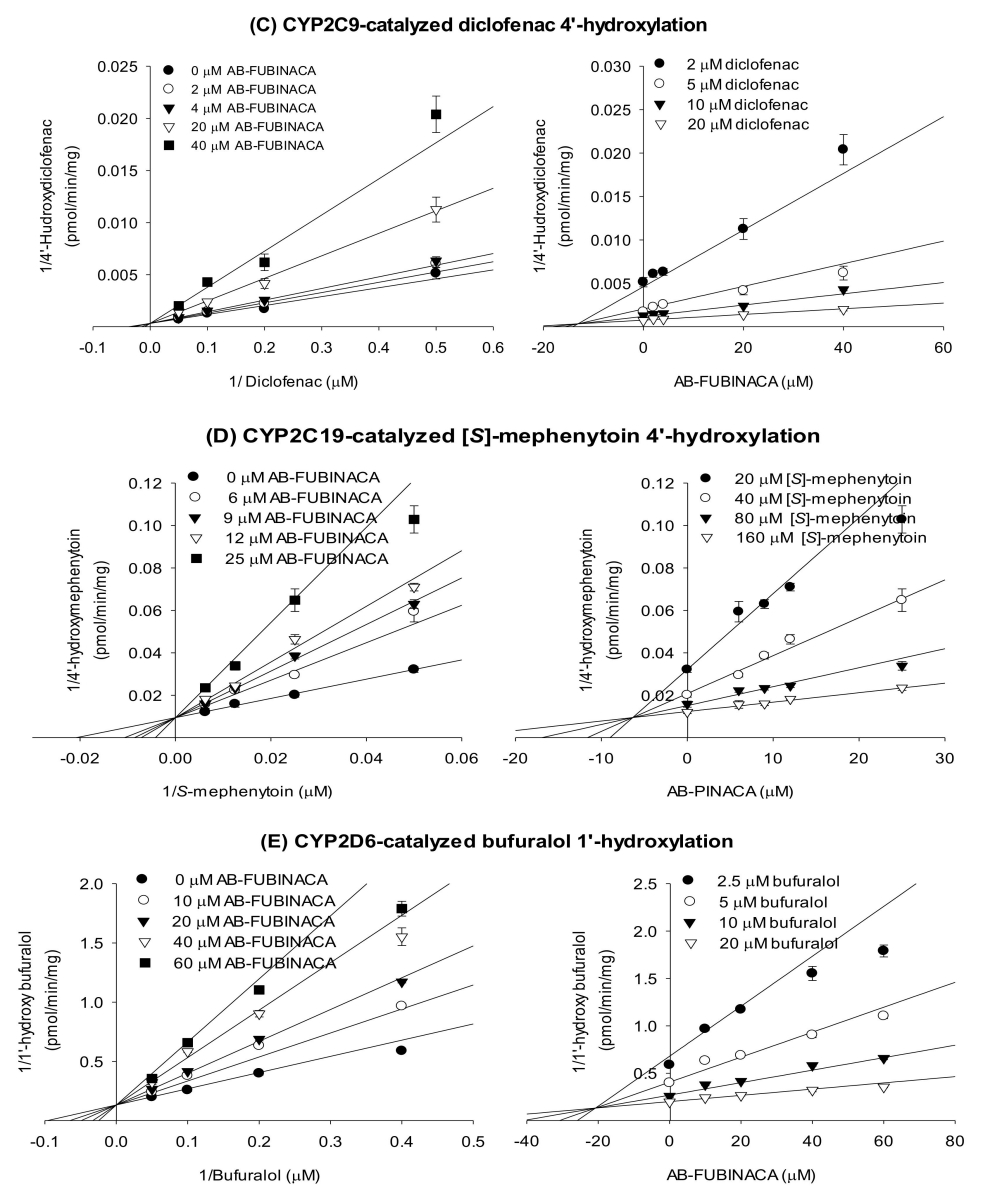
Lineweaver–Burk (left panel) and Dixon (right panel) plots of the inhibitory effects of AB-FUBINACA on (**A**) bupropion 1′-hydroxylase (CYP2B6), (**B**) amodiaquine *N*-de-ethylase (CYP2C8), (**C**) diclofenac 4′-hydroxylase (CYP2C9), (**D**) [*S*]-mephenytoin 4′-hydroxylase (CYP2C19), and (**E**) bufuralol 1′-hydroxylase (CYP2D6) of ultrapooled human liver microsomes. The data are means ± SDs (*n* = 3).

The enzyme kinetic study revealed that AB-FUBINACA competitively inhibited amodiaquine *N*-de-ethylase, diclofenac 4′-hydroxylase, [*S*]-mephenytoin 4′-hydroxylase, and bufuralol 1′-hydroxylase activities with *K_i_* values of 19.9, 13.1, 6.3, and 20.8 μM, respectively, but exhibited mixed inhibition of bupropion hydroxylase of ultrapooled human liver microsomes with *K_i_* value of 15.0 μM (Figure 3 and Table 2).

**Figure 4:**
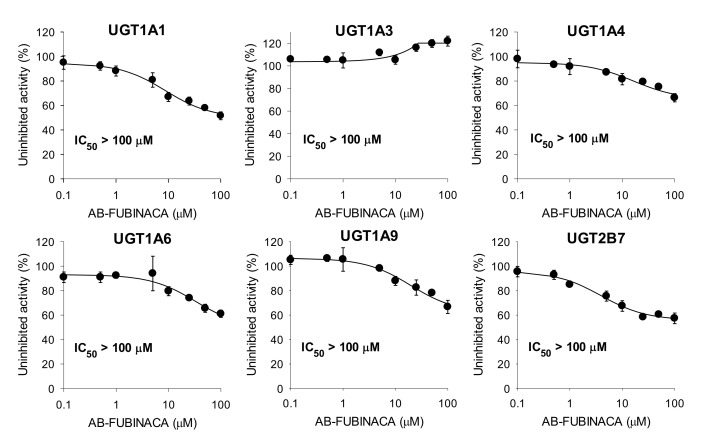
Inhibitory effects of AB-FUBINACA on uridine 5′-diphospho-glucuronosyltransferase (UGT)1A1-catalyzed SN-38 glucuronidation, UGT1A3-catalyzed chenodeoxycholic acid 24-acyl-β-glucuronidation, UGT1A4-catalyzed trifluoperazine *N*-glucuronidation, UGT1A6-catalyzed *N*-acetylserotonin glucuronidation, UGT1A9-catalyzed mycophenolic acid glucuronidation, and UGT2B7-catalyzed naloxone 3-β-D-glucuronidation by ultrapooled human liver microsomes. The concentrations of cocktail UGT substrates were 0.5 µM SN-38, 2 µM chenodeoxycholic acid, 0.5 µM trifluoperazine, 1 µM *N*-acetylserotonin, 0.2 µM mycophenolic acid, and 1 µM naloxone. The data are means ± SDs (*n* = 3).

AB-FUBINACA at 100 μM negligibly inhibited UGT1A1-catalyzed SN-38 glucuronidation, UGT1A3-catalyzed chenodeoxycholic acid 24-acyl-β-glucuronidation, UGT1A4-catalyzed trifluoperazine *N*-glucuronidation, UGT1A6-catalyzed *N*-acetylserotonin glucuronidation, UGT1A9-catalyzed mycophenolic acid glucuronidation, and UGT2B7-catalyzed naloxone 3-β-D-glucuronidation by human liver microsomes (Figure 4).

### 2.2. Inhibitory Effects of AB-FUBINACA on Drug Transporters

**Figure 5:**
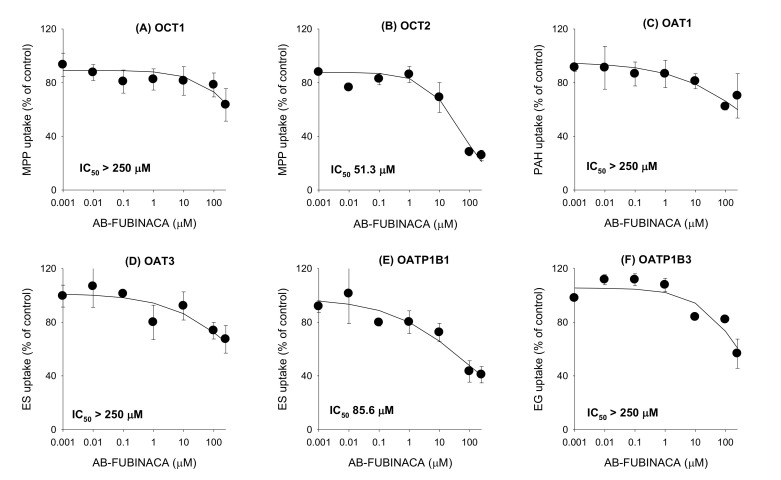
The inhibitory effects of AB-FUBINACA (0.001–250 μM) on the uptake of the probe substrate by (**A**) organic cation transporter (OCT)1, (**B**) OCT2, (**C**) organic anion transporter (OAT)1, (**D**) OAT3, (**E**) OATP1B1, and (**F**) OATP1B3 transporters. The concentrations and probe substrates were as follows: 0.1 μM [3-H]methyl-4-phenylpyridinium (for OCT1 and OCT2), 0.1 μM [3-H]para-aminohippuric acid (for OAT1), 0.1 μM [3-H]estrone-3-sulfate (ES) (for OAT3 and OATP1B1), 0.1 μM [3-H]estradiol-17β-D-glucuronide (for OATP1B3). Transporter-mediated uptake of probe substrate was calculated by subtracting the uptake of probe substrate in HEK293-mock cells from the uptake of probe substrate in HEK293 cells expressing respective transporters. The data are means ± SDs (*n* = 3).

The inhibitory effects of AB-FUBINACA on eight major transporters were evaluated using mammalian cells overexpressing OCT1, OCT2, OAT1, OAT3, OATP1B1, OATP1B3, P-gp, and BCRP. As shown in Figure 5, AB-FUBINACA inhibited OCT2-mediated MPP^+^ uptake and OATP1B1-mediated estrone-3-sulfate (ES) uptake in a concentration-dependent manner; the inhibition curves yielded IC_50_ values of 51.3 μM and 85.6 μM, respectively. AB-FUBINACA did not significantly inhibit the transport activities of OCT1, OAT1, OAT3, and OATP1B3 over the concentration ranges tested (Figure 5).

**Figure 6:**
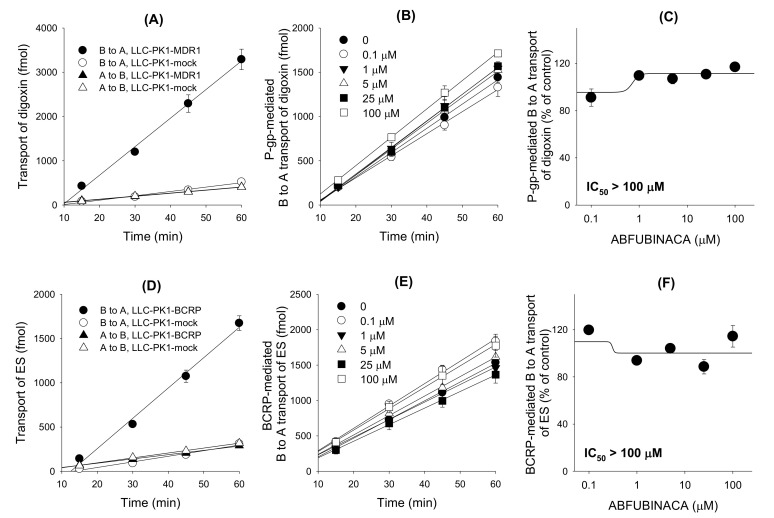
The inhibitory effects of AB-FUBINACA (0.1–100 μM) on the P-glycoprotein (P-gp)- and breast cancer resistance protein (BCRP)-mediated basal to apical (B to A) transport rate of digoxin and estrone-3-sulfate (ES), respectively. (**A**) B to A and A to B transport of 0.1 μM [3-H]digoxin was measured for 60 min in LLC-PK1-MDR1 cells and LLC-PK1-mock cells. (**B**) P-gp-mediated B to A transport of [3-H]digoxin was measure for 60 min in the presence of AB-FUBINACA (0, 0.1, 1, 5, 25 and 100 μM). P-gp-mediated B to A transport of digoxin was calculated by subtracting the B to A transport of digoxin in LLC-PK1-mock cells from the B to A transport of digoxin in LLC-PK1-MDR1 cells. (**C**) Effect of AB-FUBINACA on the P-gp-mediated B to A transport rate of digoxin was shown in a concentration range of 0.1–100 μM AB-FUBINACA. (**D**) B to A and A to B transport of 0.1 μM [3-H]ES was measured for 60 min in LLC-PK1-BCRP cells and LLC-PK1-mock cells. (**E**) BCRP-mediated B to A transport of [3-H]ES was measured for 60 min in the presence of AB-FUBINACA (0, 0.1, 1, 5, 25, 100 μM). BCRP-mediated B to A transport of ES was calculated by subtracting the B to A transport of ES in LLC-PK1-mock cells from the B to A transport of ES in LLC-PK1-BCRP cells. (**F**) Effect of AB-FUBINACA on the BCRP-mediated B to A transport rate of ES was shown in a concentration range of 0.1–100 μM AB-FUBINACA. The data are means ± SDs (*n* = 3).

The B to A transport rate of digoxin and ES, which was calculated as the slope of the graph, in LLC-PK1-MDR1 and LLC-PK1-BCRP cells was 6.5-fold and 5.4-fold, respectively, greater than that in LLC-PK1-mock cells. Net efflux ratios of digoxin and ES were 6.6 and 6.2, respectively, in LLC-PK1-MDR1 and -BCRP cells compared with LLC-PK1-mock cells (Figure 6A,D). The results suggest the feasibility of P-gp- and BCRP-mediated transport system. The effect of AB-FUBINACA on the P-gp- and BCRP-mediated B to A transport of digoxin and ES, respectively, was measured in a concentration range of 0.1–100 μM of AB-FUBINACA (Figure 6B,E) to calculate the IC_50_ value of AB-FUBINACA for P-gp and BCRP. As results, AB-FUBINACA did not inhibit the P-gp-mediated B to A transport rate of digoxin (Figure 6C) and the BCRP-mediated B to A transport rate of ES (Figure 6F) over the concentration ranges tested.

**Figure 7:**
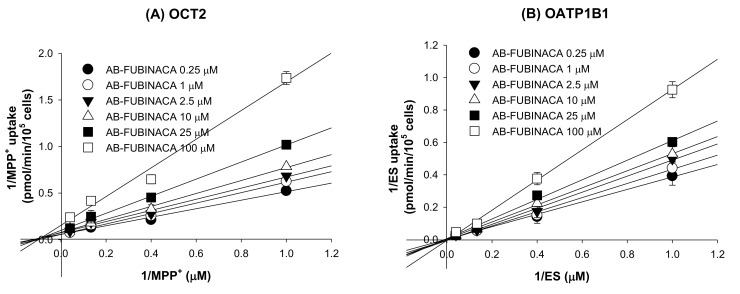
Lineweaver–Burk plots of the inhibitory effects of AB-FUBINACA (0.25–100 μM) on (**A**) OCT2-mediated [^3^H]MPP^+^ uptake and (**B**) OATP1B1-mediated ES uptake by HEK293-OCT2 and -OATP1B1 cells, respectively, in the presence of various concentrations of [^3^H]MPP^+^ or [^3^H]ES (1, 2.5, 7.5, and 25 μM). Each symbol represents a concentration of AB-FUBINACA. The data are means ± SDs (*n* = 3).

Of the eight transporters tested, the OCT2 and OATP1B1 transporters (inhibited by AB-FUBINACA) were subjected to enzyme kinetic studies to determine the modes of inhibition and the *K_i_* values. A Lineweaver–Burk plot of the inhibitory effect of AB-FUBINACA on OCT2-mediated MPP^+^ uptake revealed mixed inhibition and a *K_i_* value of 54.2 µM (Figure 7A and Table 3). AB-FUBINACA competitively inhibited OATP1B1-mediated ES uptake with a *K_i_* value of 94.4 µM (Figure 7B and Table 3).

## 3. Discussion

AB-FUBINACA competitively inhibited CYP2C8-catalyzed amodiaquine *N*-de-ethylation and CYP2C9-mediated diclofenac 4′-hydroxylation with *K_i_* values of 19.9 and 13.1 μM, respectively: other synthetic cannabinoids including AM-2201, EAM-2201, and MAM-2201 potently inhibited CYP2C8-catalyzed amodiaquine *N*-de-ethylation (*K*_i_, 0.54–2.1 µM) and CYP2C9-mediated diclofenac 4′-hydroxylation (*K*_i_, 3.0–5.6 µM) by ultrapooled human liver microsomes [27,28,29]. AB-FUBINACA exhibited mixed inhibition of CYP2B6-mediated bupropion hydroxylation with a *K_i_* of 15.0 μM. THC, cannabidiol, and cannabinol inhibited 7-benzoxyresorufin *O*-debenzylase activity in the mixed mode with *K*_i_ values of 2.81, 0.694, and 2.55 μM, respectively, as revealed by the recombinant human CYP2B6 enzyme assay [21]. AB-FUBINACA competitively inhibited CYP2C19-mediated [*S*]-mephenytoin 4′-hydroxylation with a *K_i_* of 6.3 μM (Figure 3), but EAM-2201 time-dependently inhibited CYP2C19-mediated [*S*]-mephenytoin 4′-hydroxylation by human liver microsomes with *K_i_* value of 3.8 μM and *k_inact_* of 0.0264 min^−1^ [29]. AB-FUBINACA competitively inhibited CYP2D6-mediated bufuralol 1′-hydroxylation with a *K_i_* value of 20.8 μM, and cannabidiol inhibited CYP2D6-catalyzed dextromethorphan *O*-demethylation by human liver microsomes with a *K_i_* value of 2.42 μM [22].

For accurate clinical prediction of AB-FUBINACA-induced DDI using in vitro data, the AB-FUBINACA pharmacokinetics in humans (including plasma concentrations, extents of protein binding, and tissue distributions) are necessary. However, none of AB-FUBINACA absorption, distribution, or excretion has been studied in humans or animals. A few reports on AB-FUBINACA concentrations in blood, urine, and oral fluids of drug abusers have appeared; the serum AB-FUBINACA concentration was 15 nM in a healthy 24-year-old man after ingestion of two drops of e-cigarette fluid containing AB-FUBINACA [35] and the post-mortem level of AB-FUBINACA in the femoral blood of a 35-year-old man who engaged in polysubstance abuse was 5.4 nM [36]. The concentrations of AB-FUBINACA in 5 of 70 oral fluid samples collected from 13 subjects were 2.2–106 nM [37]. The extent of AB-FUBINACA (10 μM) binding to plasma proteins was 99.7 ± 0.03% in our laboratory. Using the basic model of reversible CYP inhibition [38], the predicted ratio of the drug area under the plasma concentration curve (AUC) was less than 1.001. As an AUC change predictive of a significant DDI should exceed 1.02 according to the FDA guideline [34], AB-FUBINCA may not engage in clinically significant DDIs mediated by CYP2B6, CYP2C8, CYP2C9, CYP2C19, or CYP2D6; all were reversibly inhibited by AB-FUBINACA.

AM-2201 (*K*_i_, 4.0 µM), MAM-2201 (*K*_i_, 5.4 µM), EAM-2201 (*K_i_*_,_ 4.1 μM and *k_inact_*, 0.0250 min^−1^), and APINACA (*K_i_*, 4.5 µM; *k_inact_*, 0.04686 min^−1^) inhibited CYP3A4-catalyzed midazolam 1′-hydroxylation in human liver microsomes [27,28,29,30], but AB-FUBINACA did not inhibit the CYP3A4, CYP1A2, and CYP2A6 activities of such microsomes (Figure 2).

AB-FUBINACA negligibly inhibited the UGT1A1, UGT1A3, UGT1A4, UGT1A6, UGT1A9, and UGT2B7 activities of human liver microsomes, suggesting that the risk of clinical DDIs between AB-FUBINACA and the UGT1A1, UGT1A3, UGT1A4, UGT1A6, UGT1A9, and UGT2B7 metabolizing enzymes is remote. However, EAM-2201, MAM-2201, and AM-2201 competitively inhibited UGT1A3-mediated chenodeoxycholic acid 24-acyl-β-glucuronidation with *K_i_* values of 2.7, 4.3, and 5.0 μM, respectively, and APINACA noncompetitively inhibited UGT1A9-mediated mycophenolic acid glucuronidation (*K*_i_, 5.9 µM) by human liver microsomes [27,28,29,30].

AB-FUBINACA weakly inhibited OCT2 and OATP1B1 activities with *K*_i_ values of 54.2 and 94.4 μM, respectively; it poorly inhibited OAT1, OAT3, OCT1, and OATP1B3 activities to 250 μM; and did not affect the efflux of transporters, P-gp and BCRP to 100 μM. Thus, AB-FUBINACA is at low risk of interaction with these clinically important drug transporters; AB-FUBINACA present in the blood after drug abuse may not potentiate transporter-mediated toxicity or cause an adverse event.

## 4. Materials and Methods

### 4.1. Materials

AB-FUBINACA was obtained from Cayman Chemical Company (Ann Arbor, MI, USA). Acetaminophen, *N*-acetylserotonin, alamethicin, chenodeoxycholic acid, coumarin, 7-hydroxycoumarin, magnolol, midazolam, mycophenolic acid, naloxone, naloxone 3-β-D-glucuronide, NADPH, phenacetin, quindine, rifampin, trifluoperazine, Trizma base, uridine 5′-diphosphoglucuronoic acid (UDPGA), verapamil, sodium dodecyl sulfate (SDS), and Hank’s balanced salt solution (HBSS) were the products of Sigma-Aldrich (St. Louis, MO, USA). ^13^C_2_,^15^N-acetaminophen, bufuralol, *N*-desethylamodiaquine, 1′-hydroxybufuralol, d_9_-1′-hydroxybufuralol, 4′-hydroxydiclofenac, 4′-hydroxymephenytoin, 1′-hydroxymidazolam, [*S*]-mephenytoin, Dulbecco’s modified Eagle’s medium (DMEM), medium 199, fetal bovine serum (FBS), collagen-coated 24-transwell plates, poly-D-lysine-coated 96-well plates, LLC-PK1-MDR1 cells (LLC-PK1 cells stably expressing P-gp), LLC-PK1-mock cells, HEK293 cells transiently overexpressing the OCT1, OCT2, OAT1, OAT3, OATP1B1, and OATP1B3 transporters (HEK293-OCT1, -OCT2, -OAT1, -OAT3, -OATP1B1, and -OATP1B3 cells, respectively), and HEK293-mock cells were purchased from Corning Life Sciences (Woburn, MA, USA). LLC-PK1-BCRP cells (LLC-PK1 cells stably expressing BCRP) were obtained from Dr. A.H. Schinkel (Netherlands Cancer Institute, Amsterdam, the Netherlands). [^3^H]Methyl-4-phenylpyridinium (2.9 TBq/mmol), [^3^H]para-aminohippuric acid (0.13 TBq/mmol), [^3^H]estrone-3-sulfate (2.12 TBq/mmol), [^3^H]estradiol-17β-D-glucuronide (2.22 TBq/mmol), [^3^H]digoxin (1.103 TBq/mmol), and Optiphase scintillation cocktail were purchased from Perkin Elmer Inc. (Boston, MA, USA). *N*-acetylserotonin β-D-glucuronide, chenodeoxycholic acid 24-acyl-β-glucuronide, diclofenac, mycophenolic acid β-D-glucuronide, and SN-38 glucuronide were obtained from Toronto Research Chemicals (Toronto, ON, Canada). SN-38 was the product of Santa Cruz Biotechnology (Dallas, TX, USA). Acetonitrile, methanol, and water (LC-MS grade) were obtained from Fisher Scientific Co. (Fair Lawn, NJ, USA). All other chemicals were of the highest quality available.

### 4.2. Inhibitory Effect of AB-FUBINACA on Eight Major CYP Activities in Human Liver Microsomes

The inhibitory potentials (IC_50_ values) of AB-FUBINACA on CYP 1A2, 2A6, 2B6, 2C8, 2C9, 2C19, 2D6, and 3A4 activities in pooled human liver microsomes were evaluated using a slight modification of our previously described method that employed a cocktail of CYP substrates, followed by LC-MS/MS [27,28,29,30]. The incubation mixtures were prepared in total volumes of 100 μL as follows: 50 mM potassium phosphate buffer (pH 7.4), 1.0 mM NADPH, 10 mM MgCl_2_, ultrapooled human liver microsomes (0.2 mg/mL), various concentrations of AB-FUBINACA (final concentrations of 0.1–100 μM), and the CYP enzyme-specific substrates of the cocktail sets A (2.0 μM amodiaquine, 5 μM bufuralol, 2.5 μM coumarin, 10 μM diclofenac, 100 μM [*S*]-mephenytoin, 2.5 μM midazolam, and 50 μM phenacetin) and B (50 μM bupropion). After 3 min of pre-incubation at 37 °C, the reaction mixtures were incubated for 15 min at 37 °C after addition of NADPH in a shaking water bath. Each reaction was stopped by adding 100 μL of ice-cold methanol containing d_9_-1′-hydroxybufuralol (internal standard (IS) for 1-hydroxybupropion, 1′-hydroxybufuralol, 4′-hydroxydiclofenac, 7-hydroxycoumarin, 1′-hydroxymidazolam, and 4′-hydroxymephenytoin) and ^13^C_2_,^15^N-acetaminophen (IS for acetaminophen and *N*-desethylamodiaquine). The incubation mixtures were centrifuged at 13,000 × *g* for 8 min at 4 °C, and 50 μL of each supernatant of the A and B sets mixed. Aliquots (5 μL) of the diluted supernatants were analyzed by LC-MS/MS. All assays were performed in triplicate, and the averages were used for subsequent calculations.

To measure time-dependent inhibition, human liver microsomes were pre-incubated with various concentrations of AB-FUBINACA (final concentrations of 0.1–100 μM) and NADPH for 30 min at 37 °C. Next, the reaction mixtures were incubated with the A or B set of CYP substrates for 15 min at 37 °C. The control reaction featured the addition of methanol rather than AB-FUBINACA.

An Agilent 6495 triple quadrupole mass spectrometer coupled with an Agilent 1290 Infinity system (Agilent Technologies, Wilmington, DE, USA) was used for LC-MS/MS. Metabolites of the eight CYP substrates were simultaneously separated on an Atlantis dC18 column (3 µm, 2.1 mm internal diameter × 100 mm; Waters Co., Milford, MA, USA) using a gradient elution of 5% methanol in 0.1% formic acid (MP A) and 95% methanol in 0.1% formic acid (MP B) at a flow rate of 0.3 mL/min: 20% MP B for 0.5 min; 20–95% MP B for 0.5 min; 95% MP B for 6 min; 95–20% MP B for 0.1 min; and 20% MP B for 3 min. The column and autosampler temperatures were 40 and 4 °C, respectively. The electrospray ionization (ESI) source settings in the positive ion mode were: gas temperature, 200 °C; gas flow, 14 L/min; nebulizer pressure, 40 psi; sheath gas temperature, 380 °C; sheath gas flow, 11 L/min; capillary voltage, 4500 V; and nozzle voltage, 500 V. Quantification of each metabolite was performed in the selected reaction monitoring (SRM) mode: acetaminophen, *m*/*z* 152.1→110.1; 7-hydroxycoumarin, *m*/*z* 163.0→107.0; 4-hydroxybupropion, *m*/*z* 256.1→238.0; *N*-desethylamodiaquine, *m*/*z* 328.1→283.0; 4′-hydroxydiclofenac, *m*/*z* 312.0→231.0; 4′-hydroxymephenytoin, *m*/*z* 235.2→150.0; 1′-hydroxybufuralol, *m/z* 278.3→187.0; 1′-hydroxymidazolam, *m*/*z* 342.1→324.1, ^13^C_2_, ^15^N-acetaminophen (IS), *m*/*z* 155.1→111.1 and d_9_-1′-hydroxybufuralol (IS), *m*/*z* 287.0→187.0. The data were processed using MassHunter software ver. B.07.00 (Agilent Technologies). Typical SRM chromatograms and method validation data of eight CYP metabolites are shown in Supplementary Figure S1 and Table S1, respectively.

### 4.3. Inhibitory Effects of AB-FUBINACA on Six Major UGT Activities

The inhibitory effects of AB-FUBINACA on UGT1A1, UGT1A3, UGT1A4, UGT1A6, UGT1A9, and UGT2B7 were evaluated using our previously described LC-MS/MS method after incubation of ultrapooled human liver microsomes with a cocktail of UGT substrates [27,28,29,30]. Each incubation mixture was prepared in a final volume of 100 μL as follows: ultrapooled human liver microsomes (0.2 mg/mL), 5 mM UDPGA, 10 mM magnesium chloride, alamethicin (25 μg/mL), 50 mM Tris buffer (pH 7.4), various concentrations of AB-FUBINACA in methanol (final concentrations of 0.1–100 μM, methanol <0.5% *v*/*v*) and the UGT enzyme-specific substrates of the cocktail sets A (0.5 μM SN-38, 2 μM chenodeoxycholic acid, and 0.5 μM trifluoperazine) and B (1 μM *N*-acetylserotonin, 0.2 μM mycophenolic acid, and 1 μM naloxone). The reactions were initiated by adding UDPGA, and incubations continued for 60 min at 37 °C in a shaking water bath. The reactions were terminated by adding 50 μL of ice-cold acetonitrile containing propofol glucuronide (IS for chenodeoxycholic acid 24-acyl-β-glucuronide and mycophenolic acid glucuronide) and meloxicam (IS for SN-38 glucuronide, trifluoperazine glucuronide, *N*-acetylserotonin β-D-glucuronide, and naloxone 3-β-D-glucuronide). The incubation mixtures were centrifuged at 13,000 × *g* for 8 min at 4 °C. Then, 50 μL-aliquots of each supernatant of the A and B set were mixed and aliquots (5 μL) subjected to LC-MS/MS. All assays were performed in triplicate, and the averages were used in calculations.

The six UGT metabolites were simultaneously separated on an Atlantis dC18 column (3 µm, 2.1 mm internal diameter × 100 mm) via gradient elution of 5% acetonitrile in 0.1% formic acid (MP A) and 95% acetonitrile in 0.1% formic acid (MP B) at a flow rate of 0.3 mL/min: 10% MP B for 1 min; 10–50% MP B for 1 min; 50–95% MP B for 1 min; 95% MP B for 2 min; 95–5% MP B for 0.1 min; and 5% MP B for 2.9 min. The ESI source settings in both the positive and negative ion modes were: gas temperature, 200 °C; gas flow, 14 L/min; nebulizer pressure, 40 psi; sheath gas temperature, 380 °C; sheath gas flow, 11 L/min; capillary voltage, 4500 V; and nozzle voltage, 500 V. Each metabolite was quantified via SRM in the negative ion mode (chenodeoxycholic acid 24-acyl-β-glucuronide, *m/z* 567.1→391.2; mycophenolic acid glucuronide, *m/z* 495.0→319.0; propofol glucuronide (IS), *m/z* 353.0→177.0) and in the positive ion mode (SN-38 glucuronide, *m/z* 568.9→392.9; trifluoperazine glucuronide, *m/z* 583.9→407.9; *N*-acetylserotonin β-d-glucuronide, *m/z* 394.9→219.0; naloxone 3-β-d-glucuronide, *m/z* 503.9→309.9; meloxicam (IS), *m/z* 351.9→115.0). Typical SRM chromatograms and method validation data of six UGT metabolites are shown in Supplementary Figure S2 and Table S1, respectively.

### 4.4. Enzyme Kinetic Analysis of the Inhibition by AB-FUBINACA of Five CYP Enzyme Activities in Human Liver Microsomes

To determine the enzyme kinetic parameters and modes of inhibition of CYP2B6, CYP2C8, CYP2C9, CYP2C19, and CYP2D6 by AB-FUBINACA, various concentrations of AB-FUBINACA (final concentrations of 2–60 μM) and bupropion (final concentrations of 10–100 μM for CYP2B6), amodiaquine (1–8 μM for CYP2C8), diclofenac (2–20 μM for CYP2C9), [*S*]-mephenytoin (20–160 μM for CYP2C19), and bufuralol (2.5–20 μM for CYP2D6) were incubated with human liver microsomes (0.15 mg/mL), 10 mM MgCl_2_, 5 mM NADPH, and 50 mM phosphate buffer (pH 7.4) in a total volume of 100 μL for 30 min at 37 °C. The reaction was stopped by adding 100 μL of ice-cold acetonitrile containing d_9_-1′-hydroxybufuralol (IS for 1-hydroxybupropion, 1′-hydroxybufuralol, 4′-hydroxydiclofenac, and 4′-hydroxymephenytoin) or ^13^C_2_,^15^N-acetaminophen (IS for *N*-desethylamodiaquine), and the mixtures centrifuged at 13,000 × *g* for 4 min. Next, 50 μL of each supernatant was diluted with 50 μL of water, and aliquots (5 μL) analyzed by LC-MS/MS.

### 4.5. Inhibitory Effects of AB-FUBINACA on the Transport Activities of OCTs, OATs, OATPs, P-gp, and BCRP

HEK293 cells overexpressing OCT1, OCT2, OAT1, OAT3, OATP1B1, and OATP1B3 transporters and HEK293-mock cells were seeded into poly-D-lysine-coated 96-well plates at 10^5^ cells/well and cultured in DMEM supplemented with 10% FBS, 5 mM nonessential amino acids, and 2 mM sodium butyrate for 24 h at 37 °C in a humidified atmosphere under 8% CO_2_. After 24 h, the medium was discarded and the attached cells were washed with pre-warmed HBSS and pre-incubated for 10 min in pre-warmed HBSS at 37 °C.

To examine the effects of AB-FUBINACA on the activities of six uptake transporters, the probe uptakes into HEK293 cells overexpressing the transporters were measured in the presence of AB-FUBINACA (0–250 μM) for 5 min. The concentrations and probe substrates were as follows: 0.1 μM [^3^H]MPP^+^ (for OCT1 and OCT2), 0.1 μM [^3^H]PAH (for OAT1), 0.1 μM [^3^H]ES (for OAT3 and OATP1B1), and 0.1 μM [^3^H]EG (for OATP1B3). After 5 min, the incubation medium was discarded and the attached cells were washed three-times with ice-cold HBSS (200 μL each time) and lysed with 10% SDS solution (50 μL). The lysates were mixed thoroughly with Optiphase scintillation cocktail (250 μL) and radioactivity measured using a liquid scintillation counter. The uptake of probe into HEK293-mock cells was measured using the same protocol. The transporter-mediated uptake of probe was calculated by subtracting the uptake of HEK293-mock cells from the uptake of HEK293 cells overexpressing the OCT1, OCT2, OAT1, OAT3, OATP1B1, and OATP1B3 transporters.

LLC-PK1-MDR1, LLC-PK1-BCRP, and LLC-PK1-mock cells were grown in tissue culture flasks in medium 199 supplemented with 10% FBS and 50 μg/mL gentamycin, seeded into 24-well transwell plates at 10^5^ cells/well for 5 days to TEER values over 450 Ω·cm^2^. The B to A transport of digoxin by LLC-PK1-MDR1 and -mock cells was initiated by adding 0.8 mL of HBSS containing 0.1 μM [^3^H]digoxin and AB-FUBINACA (0–100 μM) on the basal side and 0.4 mL of fresh HBSS on the apical side of the transwell. Every 15 min for 1 h, 0.3 mL aliquots were taken from the apical side and 0.3 mL fresh prewarmed HBSS added. The B to A transport of ES in LLC-PK1-BCRP and -mock cells was measured using the same protocol in the presence of 0.1 μM [^3^H]ES and AB-FUBINACA (0–100 μM) on the basal side. The A to B transport of digoxin by LLC-PK1-MDR1 and -mock cells was initiated by adding 0.4 mL of HBSS containing 0.1 μM [^3^H]digoxin on the apical side and 0.8 mL of fresh HBSS on the basal side of the transwell. Every 15 min for 1 h, the transwell was moved to another well containing 0.8 mL fresh prewarmed HBSS. The A to B transport of ES by LLC-PK1-BCRP and -mock cells was measured using the same protocol in the presence of 0.1 μM [^3^H]ES on the apical side. Aliquots (100 μL) of transported samples were mixed with 200 μL of Optiphase scintillation cocktail and radioactivity was measured with a liquid scintillation counter. The B to A transports of digoxin and ES (mediated by P-gp and BCRP, respectively) were calculated by subtracting the probe transport of LLC-PK1-mock cells from those of LLC-PK1-MDR1 and -BCRP cells. The P-gp-mediated B to A transport rate of digoxin and the BCRP-mediated B to a transport rate of ES were calculated from the slope of the B to A transports of digoxin and ES versus time profile. Efflux ratio was calculated by dividing B to A transport ratio of probe substrate by A to B transport rate in LLC-PK1-MDR1, LLC-PK1-BCRP, and LLC-PK1-mock cells and net efflux ratio was calculate by dividing efflux ratio of probe substrate in LLC-PK1-MDR1 or LLC-PK1-BCRP by efflux ratio in LLC-PK1-mock cells.

### 4.6. Data Analysis

The inhibition data were fitted to an inhibitory effect model using Sigma Plot ver. 12.0 (Systat Software Inc., San Jose, CA, USA) to obtain the IC_50_ values (the half-maximal inhibitory concentrations) of AB-FUBINACA [30]. *K*_i_ (inhibition constant) values of AB-FUBINACA and the mode of inhibition were calculated/derived by drawing Lineweaver–Burk plots [39] using Enzyme Kinetics ver. 1.1 (Systat Software, Inc.).

## 5. Conclusions

The in vitro inhibitory effects of AB-FUBINACA on eight major clinically important CYP and six UGT enzymes of ultrapooled human liver microsomes and on six solute carrier transporters and two efflux transporters using a transporter expression system were investigated for the first time to predict the drug interaction potential of AB-FUBINACA via the modulation of drug-metabolizing enzymes and transporters. AB-FUBINACA moderately inhibited CYP2B6, CYP2C8, CYP2C9, CYP2C19, CYP2D6, OCT2, and OATP1B1 activities with *K*_i_ values of 15.0, 19.9, 13.1, 6.3, 20.8, 54.2, and 94.4 μM, respectively. Although in vitro inhibition of CYP, UGT, and transporter activities does not necessarily translate into significant DDIs using a basic prediction model, it is necessary to evaluate the in vivo potential of AB-FUBINACA to cause DDIs via the inhibition of CYP2B6, CYP2C8, CYP2C9, CYP2C19, CYP2D6, OCT2, and OATP1B1 activities.

## Figures and Tables

**Table 1:** Inhibitory potential of AB-FUBINACA on eight major cytochrome P450 (CYP) enzyme activities of ultrapooled human liver microsomes in the presence and absence of 30-min preincubation with NADPH.

CYPs	Enzyme Activity	IC_50_ (μM)	
		No Preincubation	With 30 min-Preincubation
1A2	Phenacetin *O*-de-ethylase	>100	>100
2A6	Coumarin 7-hydroxylase	>100	>100
2B6	Bupropion hydroxylase	29.9	63.8
2C8	Amodiaquine *N*-de-ethylase	44.3	56.2
2C9	Diclofenac 4′-hydroxylase	13.5	14.8
2C19	[*S*]-Mephenytoin 4′-hydroxylase	19.7	18.5
2D6	Bufuralol 1′-hydroxylase	40.4	96.8
3A4	Midazolam 1′-hydroxylase	>100	>100

The data represent the average of three measurements.

**Table 2:** Enzyme kinetic parameters revealing the inhibitory potential of AB-FUBINACA in terms of the CYP and drug transporter activities of pooled human liver microsomes.

CYPs	Enzyme Activity	*K*_i_ (μM)	Inhibition Mode
2B6	Bupropion hydroxylase	15.0	Mixed
2C8	Amodiaquine *N*-de-ethylase	19.9	Competitive
2C9	Diclofenac 4′-hydroxylase	13.1	Competitive
2C19	[*S*]-Mephenytoin 4′-hydroxylase	6.3	Competitive
2D6	Bufuralol 1′-hydroxylase	20.8	Competitive

**Table 3:** Enzyme kinetic parameters reflecting the inhibitory potential of AB-FUBINACA in the context of drug transporters.

Transporter	*K*_i_ (μM)	Inhibition Mode
OCT2	54.2	Mixed
OATP1B1	94.4	Competitive

## Supplementary Materials

The following are available online, Figure S1: SRM chromatograms of CYP metabolites formed from human liver microsomal incubation of eight CYP cocktail substrates with NADPH and two IS, Figure S2: SRM chromatograms of UGT metabolites formed from human liver microsomal incubation of six UGT cocktail substrates with UDPGA and two IS, Table S1: Concentration ranges and correlation coefficients of the calibration curves and precision (coefficient of variation, CV) and accuracy values for CYP and UGT metabolites.
