# Supplementary material for: In Vitro Interaction of AB-FUBINACA with Human Cytochrome P450, UDP-Glucuronosyltransferase Enzymes and Drug Transporters
Source: Molecules. 2020 Oct 8;25(19):4589. doi: 10.3390/molecules25194589 (PMC7582776; doi:10.3390/molecules25194589)
Supplement: Supplementary file 1 [file molecules-25-04589-s001.pdf]

Supplementary Materials

In Vitro Interaction of AB-FUBINACA with Human Cytochrome P450, UDP-Glucuronosyltransferase Enzymes and Drug Transporters

Sunjoo Kim[1#], Dong Kyun Kim[1#], Yongho Shin[1], Ji-Hyeon Jeon [2], Im-Sook Song [2,*] and Hye Suk Lee [1,*]

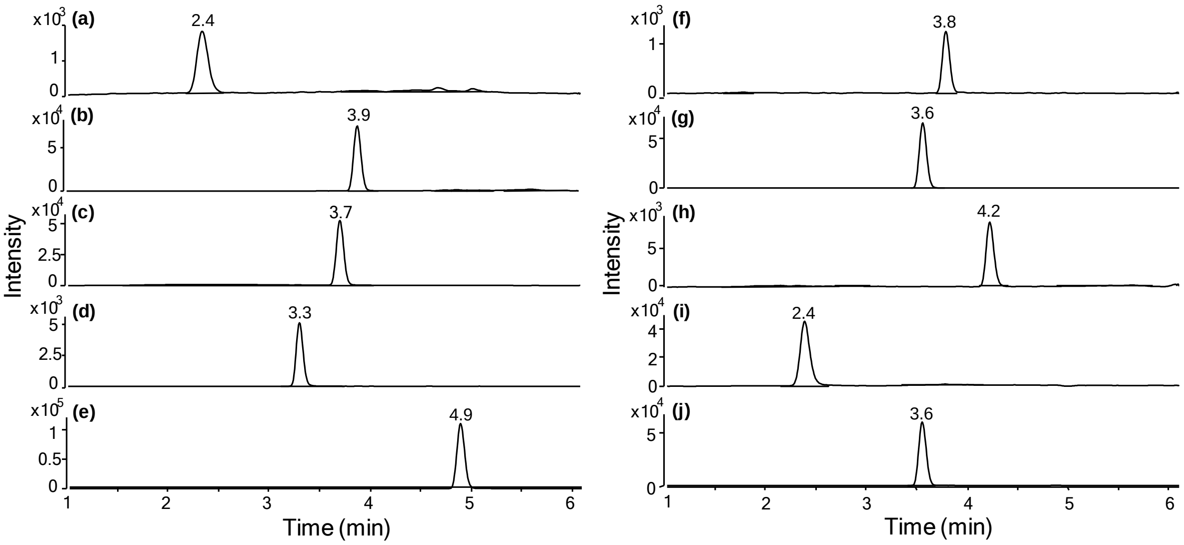

Figure S1. SRM chromatograms of CYP metabolites formed from human liver microsomal incubation of eight CYP cocktail substrates with NADPH and two IS. (a) acetaminophen, (b) 7-hydroxycoumarin, (c) hydroxybupropion, (d) *N*-desethylamodiaquine, (e) 4′-hydroxydiclofenac, (f) 4′-hydroxymephenytoin, (g) 1′-hydroxybufuralol, (h) 1′-hydroxymidazolam, (i) $^{13}C_2$,^{15}N-acetaminophen, and (j) d_9-1'-hydroxybufuralol (IS).

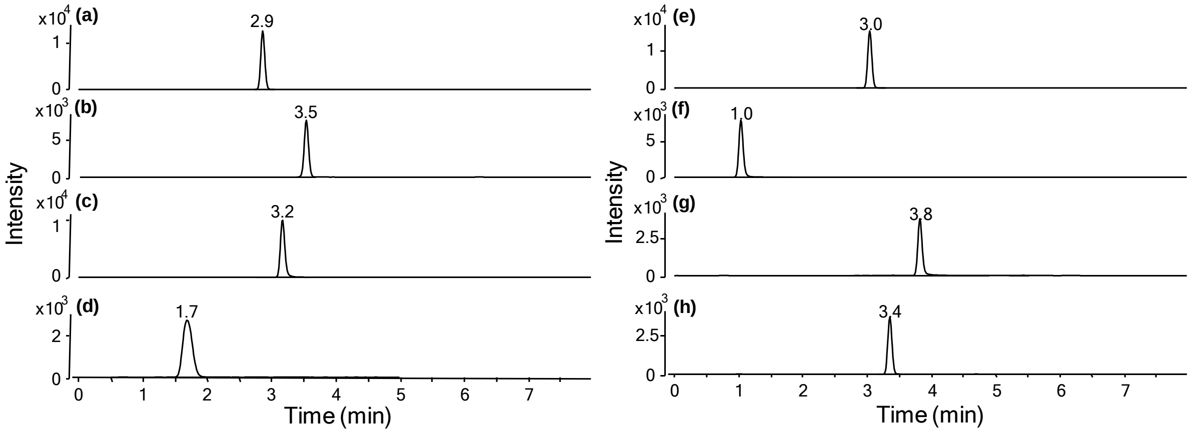

Figure S2. SRM chromatograms of UGT metabolites formed from human liver microsomal incubation of six UGT cocktail substrates with UDPGA and two IS. : (a) SN-38 glucuronide, (b) chenodeoxycholic acid 24-acyl-β-glucuronide, (c) trifluoperazine *N*-glucuronide, (d) *N*-acetylserotonin glucuronide, (e) mycophenolic acid glucuronide, (f) naloxone 3-β-D-glucuronide. (g) meloxicam (IS), and (h) propofol glucuronide (IS).

Table S1. Concentration ranges and correlation coefficients of the calibration curves and precision (coefficient of variation, CV) and accuracy values for CYP and UGT metabolites.

analytes	SRM transition (*m*/*z*) (collision energy, eV)	polarity	Concentration range (pmol)	Correlation coefficient	QC (pmol)	Intra-day (n = 5)	
						Accuracy (%)	CV (%)
acetaminophen	152.1→110.1 (8)	ESI^+	5-200	0.9971	15, 70, 140	96.8	5.4
7-hydroxycoumarin	163.0→107.0 (12)	ESI^+	15-600	0.9963	45, 120, 420	98.7	6.9
hydroxybupropion	256.1→238.0 (5)	ESI^+	1-40	0.9973	3, 8, 28	99.7	8.6
N-desethylamodiaquine	328.1→283.0 (55)	ESI^+	15-600	0.9970	45, 210, 420	95.9	8.4
4′-hydroxydiclofenac	312.0→231.0 (17)	ESI^+	1-100	0.9908	3, 20, 70	97.3	6.8
4′-hydroxymephenytoin	235.2→150.0 (8)	ESI^+	0.5-20	0.9946	1.5, 7, 14	94.8	8.2
1′-hydroxybufuralol	278.3→187.0 (8)	ESI^+	0.5-20	0.9993	1.5, 7, 14	96.9	6.3
1′-hydroxymidazolam	342.1→324.1 (8)	ESI^+	5-200	0.9981	15, 70, 140	99.6	9.0
SN-38 glucuronide	568.9→392.9 (30)	ESI^+	1-300	0.9967	3, 30, 210	105.5	5.4
chenodeoxycholic acid 24-acyl-β-glucuronide	567.1→391.2 (34)	ESI^-	1-300	0.9980	3, 30, 210	102.9	10.1
trifluoperazine *N*-glucuronide	583.9→407.9 (26)	ESI^+	4-1200	0.9998	12, 120, 840	98.8	9.9
N-acetylserotonin glucuronide	394.9→219.0 (10)	ESI^+	1-300	0.9956	3, 30, 210	97.9	6.5
mycophenolic acid glucuronide	495.0 →319.0 (20)	ESI^-	1-300	0.9958	3, 30, 210	98.1	7.9
naloxone 3-β-D-glucuronide	503.9→309.9 (32)	ESI^+	1-300	0.9971	3, 30, 210	100.3	8.4
